# Effectiveness of Resmetirom in Reducing Cholesterol Levels in Patients With Nonalcoholic Steatohepatitis: A Systematic Review and Meta-Analysis

**DOI:** 10.7759/cureus.70859

**Published:** 2024-10-04

**Authors:** Estifanos Bejitual, Muhammad Faisal Awais, Dhruvi Modi, Ushna Gul, Kinan Obeidat, Najeeha Ahmed, Muhammad Daniyal Waheed, Shamsha Hirani

**Affiliations:** 1 Internal Medicine, St. John’s Episcopal Hospital, Far Rockaway, USA; 2 Acute Medicine, Dudley NHS Trust, Dudley, GBR; 3 Internal Medicine, Gujarat Adani Institute of Medical Sciences, Bhuj, IND; 4 Internal Medicine, Khyber Medical College, Peshawar, PAK; 5 Internal Medicine, University of Texas Medical Branch at Galveston, Galveston, USA; 6 Medicine, Rawalpindi Medical University, Rawalpindi, PAK; 7 Internal Medicine, Foundation University Medical College, Islamabad, PAK; 8 Cardiology, Baqai Hospital, Karachi, PAK

**Keywords:** cholesterol levels, effectiveness, nonalcoholic steatohepatitis, resmetirom, systematic review and meta-analysis

## Abstract

Nonalcoholic steatohepatitis (NASH) is a progressive form of nonalcoholic fatty liver disease (NAFLD) associated with metabolic syndrome and increased cardiovascular risk. Resmetirom, a novel liver-directed selective thyroid hormone receptor-β (THR-β) agonist, has shown promise in addressing both hepatic and systemic lipid metabolism. This systematic review and meta-analysis aimed to evaluate the efficacy of resmetirom in improving cholesterol levels in NASH patients. A systematic literature search was conducted across multiple databases including PubMed, Embase, Cochrane Library, and ClinicalTrials.gov, identifying three randomized controlled trials for inclusion. The meta-analysis revealed that resmetirom significantly reduced low-density lipoprotein cholesterol (LDL-C) levels compared to placebo (MD: -23.62; 95% CI: -37.32 to -9.93; p < 0.001). Similarly, triglyceride (TG) levels showed a significant reduction in the resmetirom group (MD: -33.86; 95% CI: -47.79 to -19.92; p < 0.001). Importantly, there was no significant difference in the risk of serious adverse events between resmetirom and placebo groups (RR: 1.09; 95% CI: 0.73 to 1.63; p = 0.67). These findings suggest that resmetirom effectively improves lipid profiles in NASH patients without compromising safety. However, the analysis was limited by the small number of studies, all from the same research group, and high heterogeneity in results. Future research should include more diverse studies, longer follow-up periods, and cost-effectiveness evaluations. Despite these limitations, resmetirom shows promise as a potential treatment for managing dyslipidemia and cardiovascular risk in NASH patients, potentially influencing future treatment guidelines for both liver and cardiovascular health in this population.

## Introduction and background

Nonalcoholic steatohepatitis (NASH) is a progressive form of nonalcoholic fatty liver disease (NAFLD) characterized by hepatic inflammation and fibrosis [1-2]. As a major cause of liver-related morbidity and mortality, NASH has gained prominence due to its association with metabolic syndrome, insulin resistance, obesity, and particularly dyslipidemia [3]. Among these metabolic abnormalities, elevated cholesterol levels, specifically low-density lipoprotein cholesterol (LDL-C) and triglycerides (TG), play a critical role in both the progression of liver disease and the increased cardiovascular risk observed in NASH patients [4]. Cardiovascular disease remains one of the leading causes of death in individuals with NASH, underscoring the need for effective treatments that address not only liver health but also the broader metabolic complications of the disease [5]. 

Current treatment options for managing dyslipidemia in NASH patients are limited, with statins being the most widely used class of drugs [6]. However, many patients continue to experience dysregulated lipid profiles and remain at high risk for cardiovascular events despite statin therapy and lifestyle modifications [7]. This unmet need has driven research into novel therapeutic agents that can target both hepatic and systemic lipid metabolism. 

Resmetirom (MGL-3196) is a novel, liver-directed selective thyroid hormone receptor-β (THR-β) agonist. Its mechanism of action involves selectively binding to THR-β receptors in the liver, where it enhances the metabolic processes that regulate lipid and cholesterol levels. Resmetirom has demonstrated the ability to lower LDL-C, TG, and non-high-density lipoprotein cholesterol (non-HDL-C) in early-phase clinical trials [8-9]. In addition, it has shown promising effects in reducing hepatic fat and fibrosis markers in patients with NASH. By targeting both lipid abnormalities and liver pathology, resmetirom offers a dual approach that could potentially modify disease progression while also improving cardiovascular outcomes [10]. Unlike thyroid hormone therapies that can affect multiple organs and cause undesirable side effects, resmetirom's liver selectivity makes it a safer option for long-term treatment [10]. 

Despite these promising findings, the overall efficacy of resmetirom in improving cholesterol levels in NASH patients has not been systematically evaluated. This systematic review and meta-analysis aim to assess the impact of resmetirom on key lipid parameters, including LDL-C and TG. The objective is to synthesize available evidence and provide a comprehensive evaluation of resmetirom's potential to improve dyslipidemia in patients with NASH, thus helping to guide future clinical decision-making. 

## Review

Methodology 

Literature Search and Search Strategy 

A systematic literature search was conducted across multiple databases, including PubMed, Embase, Cochrane Library, and ClinicalTrials.gov, to identify relevant studies evaluating the efficacy of resmetirom in improving cholesterol levels in patients with NASH. The search strategy was developed using a combination of Medical Subject Headings (MeSH) terms and keywords related to “resmetirom,” “MGL-3196,” “NASH,” “cholesterol,” “lipid metabolism,” “LDL-C,” and “triglycerides.” Boolean operators (AND, OR) were employed to ensure a comprehensive search. No language or publication date restrictions were applied in order to capture the maximum number of relevant studies. Additionally, reference lists of included studies and related reviews were manually searched to identify any studies missed during the initial search. The search was performed by two authors (MA and DM). Disagreements between reviewers were resolved through discussion.

Study Selection 

Studies were included in the review if they met the following predefined criteria: (1) randomized controlled trials (RCTs) or observational studies that evaluated the effect of resmetirom on cholesterol levels in NASH patients; (2) studies that reported at least one lipid outcome measure, including LDL-C, or TG; and (3) studies that provided sufficient data for extraction and analysis. Articles were excluded if they were animal studies, case reports, or lacked necessary outcome data. After removing duplicates, two independent reviewers (UG and NA) screened the titles and abstracts of all identified thearticles. Full-text reviews were conducted on potentially eligible studies to confirm their inclusion based on the above criteria. Disagreements between reviewers were resolved through discussion or, when necessary, by consulting a third reviewer. 

Data Extraction, Outcome Measures, and Risk Assessment

Data extraction was performed independently by two reviewers (KO and NA) using a standardized data extraction form. The extracted data included study characteristics (author, year, design, sample size), intervention details (dose of resmetirom), and change in outcomes from baseline (LDL-C and TG). For safety, we compared the risk of serious adverse events between two groups. If necessary, corresponding authors of studies were contacted for clarification or additional data. Risk of bias was assessed using Cochrane Risk of Bias assessment tool.

Statistical Analysis 

The meta-analysis was performed using statistical software Review Manager (RevMan) (Cochrane, London, England) to pool the results of the included studies. The effect size was estimated using mean differences (MDs) for continuous outcomes, with 95% confidence intervals (CI). We put a p-value cutoff at 0.05 for statistical significance. A random-effects model was applied to account for heterogeneity among studies. Heterogeneity was assessed using the I² statistic, with values above 50% indicating substantial heterogeneity [11]. In this meta-analysis, we were not able to perform publication bias as the number of included studies was less than 10. 

Results 

**Figure 1:**
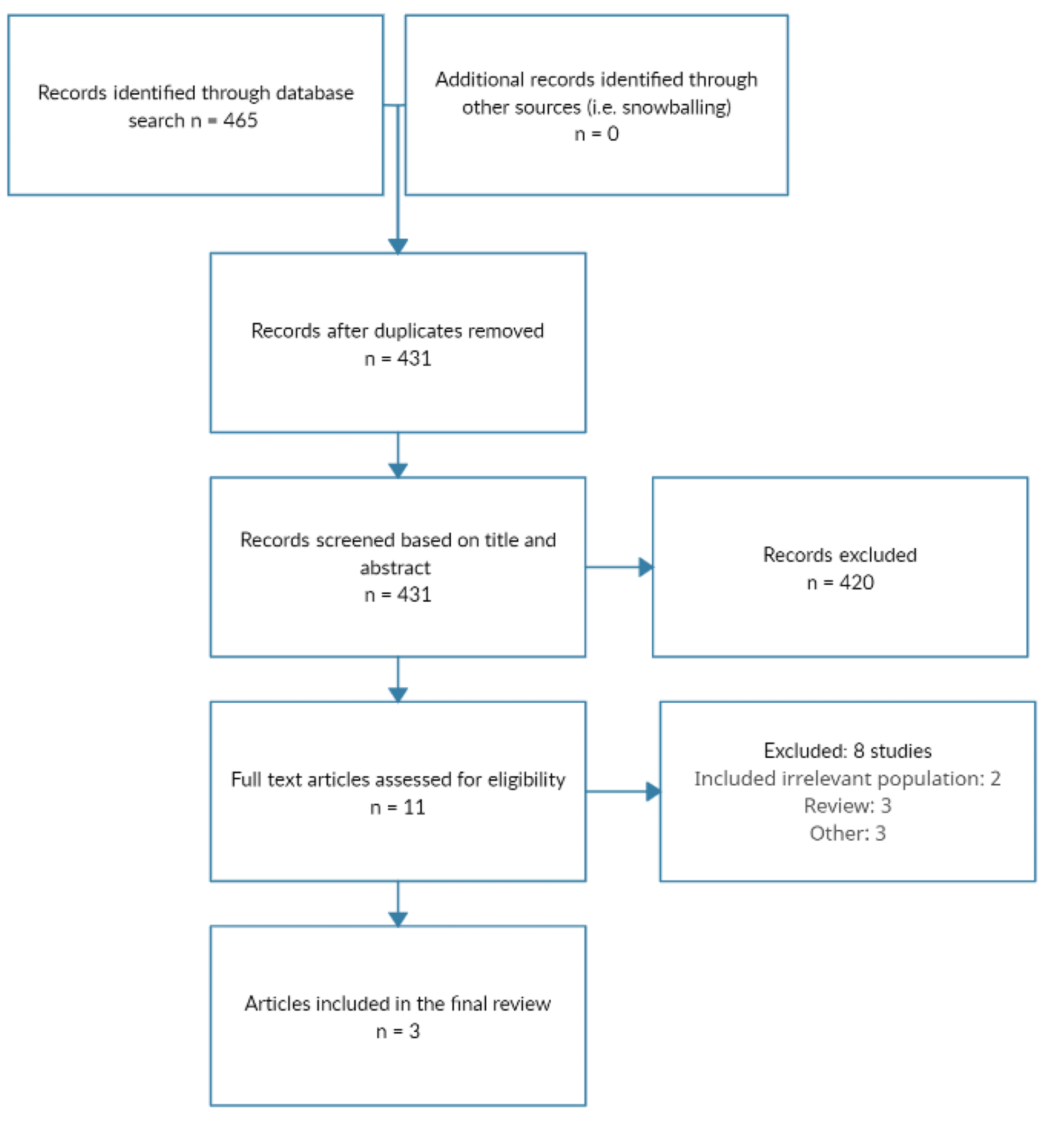
PRISMA flowchart of study selection PRISMA: Preferred Reporting Items for Systematic Reviews and Meta-Analyses

**Figure 2:**
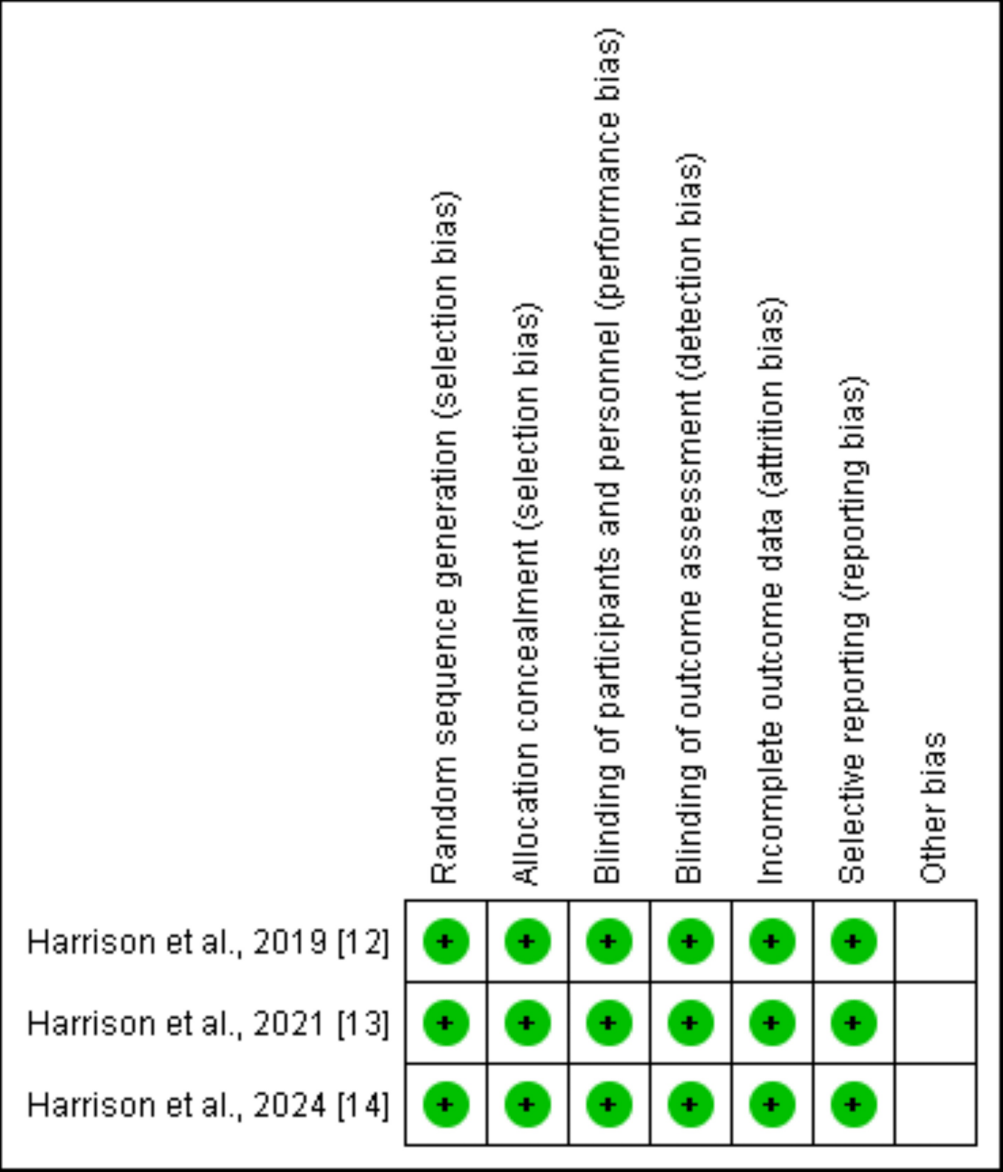
Risk-of-bias summary

Through a comprehensive search, we found 465 relevant studies. Initial screening was done after removing duplicates. After initial screening, 431 studies were removed. In the final phase of the study selection, full-text of 11 studies were obtained and detailed assessment based on predefined inclusion and exclusion criteria was performed. Finally, three studies were included in this meta-analysis. Figure 1 shows the screening and study selection process. Table 1 shows the characteristics of included studies. All studies were RCTs and included pooled sample of 799 patients with NASH. Figure 2 presents a risk-of-bias summary of the included studies.

**Table 1:** Characteristics of included studies (n = 3) RCT: Randomized controlled trial

Author name	Year	Region	Study design	Groups	Sample Size	Dose of resmetirom	Follow-up duration	Age (years)
Harrison et al. [12]	2019	United States	Phase II RCT	Resmetirom	84	80 mg	38 weeks	51.8
				Placebo	41			47.3
Harrison et al. [13]	2021	United States	Phase II RCT	Resmetirom	14	80 mg	36 weeks	53.1
				Placebo	17			42.4
Harrison et al. [14]	2024	United States	Phase III RCT	Resmetirom	322	80 mg	24 weeks	55.7
				Placebo	321			56.2

Change in LDL-C From Baseline

**Figure 3:**
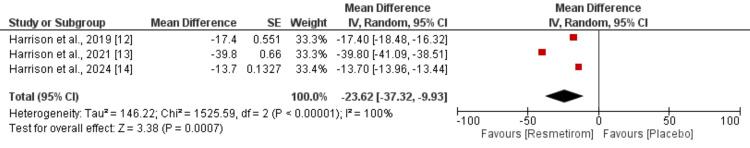
Change in LDL-C from baseline LDL-C: Low-density lipoprotein cholesterol Sources: [12-14]

All three studies evaluated the impact of resmetirom on the change in LDL-C levels from baseline, and results of the pooled analysis are shown in Figure 3. As shown in pooled analysis, the reduction in LDL-C from baseline was significantly higher in patients receiving resmetirom compared to the patients receiving placebo (MD: -23.62; 95% CI: -37.32 to -9.93; p < 0.001). High heterogeneity was reported among the study results (I-Square: 100%). All included studies reported significantly greater reduction of LDL-C in resmetirom patients. Heterogeneity among the study results was high potentially due to differences in follow-up duration.

 *Change in TG From Baseline *

**Figure 4:**
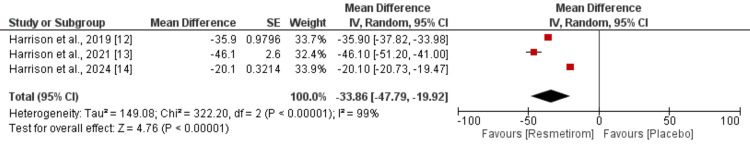
Change in triglycerides from baseline Sources: [12-14]

All three studies evaluated the impact of resmetirom on the change in TG from baseline, and results of the pooled analysis are shown in Figure 4. As shown in the pooled analysis, the reduction in TG from baseline was significantly higher in patients receiving resmetirom compared to the patients receiving placebo (MD: -33.86; 95% CI: - 47.79 to -19.92; p < 0.001). High heterogeneity was reported among the study results (I-Square: 99%). All included studies reported significantly greater reduction of TG in resmetirom patients. 

*Serious Adverse Events* 

**Figure 5:**
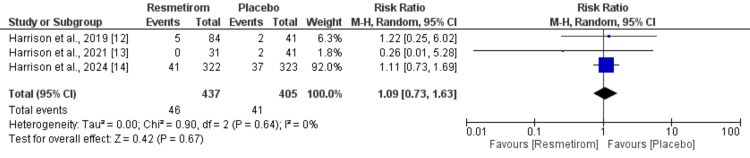
Serious adverse events between two groups Sources: [12-14]

Three studies compared the risk of serious adverse events between resmetirom and placebo groups, and the results are shown in Figure 5. Pooled analysis showed that the risk of serious adverse events was not significantly different between the resmetirom and placebo groups (RR: 1.09; 95% CI: 0.73 to 1.63; p = 0.67). No significant heterogeneity was reported among the study results.

Discussion 

This meta-analysis was conducted with the aim to compare the efficacy and safety of resmetirom in improving cholesterol levels in patients with NASH. The study found that patients receiving resmetirom showed better efficacy in reducing LDL-C and TG without affecting the safety of patients. No significant increase in adverse events was observed, indicating that resmetirom is a well-tolerated treatment for this patient population. 

Patients with NASH, many of whom also suffer from diabetes, face an elevated risk of cardiovascular diseases and mortality [15]. In this context, the notable reductions in TG and LDL-C observed with resmetirom treatment are particularly significant. These findings highlight resmetirom's potential in improving dyslipidemia and addressing metabolic imbalances linked to NASH. Additionally, the observed substantial decrease in reverse T3 (rT3) levels suggests a potential restoration of thyroid hormone equilibrium, which may contribute to alleviating metabolic disturbances [16]. 

Resmetirom demonstrates superior efficacy in reducing LDL-C and TG in patients compared to placebo due to its selective action on THR-β [17]. Unlike traditional thyroid hormone therapies that can affect various organs and potentially cause systemic side effects, resmetirom specifically targets THR-β receptors in the liver [12]. This targeted action enhances lipid metabolism by increasing the clearance of LDL-C and TG from the bloodstream [8]. Resmetirom promotes the upregulation of genes involved in fatty acid oxidation and lipoprotein catabolism, leading to more efficient breakdown of circulating lipids [11]. Additionally, resmetirom reduces the production of very-low-density lipoprotein (VLDL), a precursor of LDL-C, thereby further lowering LDL-C levels. The selective activation of THR-β also helps in reducing hepatic fat accumulation, which is crucial for improving lipid profiles in patients with NASH [18]. This focused approach not only improves lipid metabolism but also minimizes off-target effects, making resmetirom a more effective option for managing dyslipidemia in NASH patients compared to placebo [13]. 

Reducing LDL-C and TG in patients with metabolic-associated steatotic hepatopathy (MASH) offers substantial benefits. Lower LDL-C and TG levels significantly reduce cardiovascular risk, which is crucial as cardiovascular disease is prevalent among these patients [19]. Additionally, decreasing these lipids can mitigate liver inflammation and fibrosis, potentially slowing disease progression. Improved lipid profiles also enhance insulin sensitivity, helping to manage blood glucose levels and reduce the risk of type 2 diabetes [20]. Furthermore, lowering LDL-C and TG contributes to decreased hepatic fat accumulation, improving overall liver function and reducing the risk of advancing liver disease [21-22]. 

The future implications of this research into resmetirom’s effects on LDL-C and TG in patients with MASH are significant. If proven effective, resmetirom could become a pivotal treatment for managing dyslipidemia and mitigating cardiovascular risk in this population. This could lead to improved clinical outcomes and quality of life for patients with MASH. Additionally, successful results may pave the way for further research into targeted therapies for metabolic liver diseases and enhance our understanding of lipid management in complex conditions. Ultimately, this research could influence treatment guidelines and therapeutic strategies for managing both liver and cardiovascular health. 

This meta-analysis has several limitations. Firstly, it included only three studies, all of which specifically focused on patients with NASH, restricting the ability to conduct subgroup analyses. The heterogeneity in the outcomes of these studies raises concerns about potential publication bias, further exacerbated by limited access to unpublished data. Moreover, the fact that all three studies were conducted by the same research group may have increased variability in the findings. This highlights the necessity for incorporating studies from different sources to reduce biases and strengthen the reliability of results. Additionally, the analysis did not account for the effects of short follow-up durations on long-term outcomes or include a cost-effectiveness evaluation, which limits the generalizability of the findings. The lack of detailed baseline characteristics also restricts the understanding of possible influencing factors. These issues emphasize the need for careful interpretation and suggest that future research should address these gaps to improve the overall robustness of the evidence. 

## Conclusions

This meta-analysis illustrates that resmetirom profoundly improves lipid profiles in NASH patients, particularly by decreasing LDL-C and TG, without increasing the risk of serious adverse events. These outcomes propose resmetirom's potential as a successful and viable treatment for dyslipidemia in NASH, potentially addressing both liver health and cardiovascular risk. However, the analysis is limited by the small number of studies, all from the same research group, and high heterogeneity in results. Future research should include more diverse studies, lengthier follow-up intervals, and cost-effectiveness evaluations to strengthen and augment the evidence backing. Despite these limitations, resmetirom shows promise in managing the complex metabolic challenges associated with NASH.

## References

[1] Basaranoglu M, Neuschwander-Tetri BA (2006). Nonalcoholic fatty liver disease: clinical features and pathogenesis. Gastroenterol Hepatol (N Y).

[2] Loomba R, Friedman SL, Shulman GI (2021). Mechanisms and disease consequences of nonalcoholic fatty liver disease. Cell.

[3] Cariou B, Byrne CD, Loomba R, Sanyal AJ (2021). Nonalcoholic fatty liver disease as a metabolic disease in humans: a literature review. Diabetes Obes Metab.

[4] Sun DQ, Liu WY, Wu SJ (2016). Increased levels of low-density lipoprotein cholesterol within the normal range as a risk factor for nonalcoholic fatty liver disease. Oncotarget.

[5] Shroff H, VanWagner LB (2020). Cardiovascular disease in nonalcoholic steatohepatitis: screening and management. Curr Hepatol Rep.

[6] Martin A, Lang S, Goeser T, Demir M, Steffen HM, Kasper P (2022). Management of dyslipidemia in patients with non-alcoholic fatty liver disease. Curr Atheroscler Rep.

[7] Sayuti NH, Muhammad Nawawi KN, Goon JA, Mokhtar NM, Makpol S, Tan JK (2023). A review of the effects of fucoxanthin on NAFLD. Nutrients.

[8] Karim G, Bansal MB (2023). Resmetirom: an orally administered, smallmolecule, liver-directed, β-selective THR agonist for the treatment of non-alcoholic fatty liver disease and non-alcoholic steatohepatitis. touchREV Endocrinol.

[9] Kokkorakis M, Boutari C, Hill MA, Kotsis V, Loomba R, Sanyal AJ, Mantzoros CS (2024). Resmetirom, the first approved drug for the management of metabolic dysfunction-associated steatohepatitis: trials, opportunities, and challenges. Metabolism.

[10] Noureddin M, Charlton MR, Harrison SA (2024). Expert panel recommendations: practical clinical applications for initiating and monitoring resmetirom in patients with MASH/NASH and moderate to noncirrhotic advanced fibrosis. Clin Gastroenterol Hepatol.

[11] Lin L (2020). Comparison of four heterogeneity measures for meta-analysis. J Eval Clin Pract.

[12] Harrison SA, Bashir MR, Guy CD (2019). Resmetirom (MGL-3196) for the treatment of non-alcoholic steatohepatitis: a multicentre, randomised, double-blind, placebo-controlled, phase 2 trial. The Lancet.

[13] Harrison SA, Bashir M, Moussa SE, McCarty K, Pablo Frias J, Taub R, Alkhouri N (2021). Effects of resmetirom on noninvasive endpoints in a 36‐week phase 2 active treatment extension study in patients with NASH. Hepatol Commun.

[14] Harrison SA, Bedossa P, Guy CD (2024). A phase 3, randomized, controlled trial of resmetirom in NASH with liver fibrosis. N Engl J Med.

[15] Angulo P, Kleiner DE, Dam-Larsen S (2015). Liver fibrosis, but no other histologic features, is associated with long-term outcomes of patients with nonalcoholic fatty liver disease. Gastroenterology.

[16] Halsall DJ, Oddy S (2021). Clinical and laboratory aspects of 3,3',5'-triiodothyronine (reverse T3). Ann Clin Biochem.

[17] Saponaro F, Sestito S, Runfola M, Rapposelli S, Chiellini G (2020). Selective thyroid hormone receptor-beta (TRβ) agonists: New perspectives for the treatment of metabolic and neurodegenerative disorders. Front Med (Lausanne).

[18] Byrne CD, Targher G, Tilg H (2024). Thyroid hormone receptor-beta agonists: new MASLD therapies on the horizon. Gut.

[19] Gouveia R, Madureira S, Elias C (2023). Lower low density lipoprotein cholesterol associates to higher mortality in non-diabetic heart failure patients. Int J Cardiol Cardiovasc Risk Prev.

[20] Arvanitakis K, Koufakis T, Kalopitas G, Papadakos SP, Kotsa K, Germanidis G (2024). Management of type 2 diabetes in patients with compensated liver cirrhosis: short of evidence, plenty of potential. Diabetes Metab Syndr.

[21] Kim KS, Hong S, Han K, Park CY (2024). Association of non-alcoholic fatty liver disease with cardiovascular disease and all cause death in patients with type 2 diabetes mellitus: nationwide population based study. BMJ.

[22] Simon TG, Bamira DG, Chung RT, Weiner RB, Corey KE (2017). Nonalcoholic steatohepatitis is associated with cardiac remodeling and dysfunction. Obesity (Silver Spring).

